# Effect of isoniazid preventive therapy on immune responses to *mycobacterium tuberculosis*: an open label randomised, controlled, exploratory study

**DOI:** 10.1186/s12879-015-1201-8

**Published:** 2015-10-22

**Authors:** Irene Andia Biraro, Moses Egesa, Simon Kimuda, Steven G. Smith, Frederic Toulza, Jonathan Levin, Moses Joloba, Achilles Katamba, Stephen Cose, Hazel M. Dockrell, Alison M Elliott

**Affiliations:** Department of Internal Medicine, College of Health Sciences, Makerere University, P.O Box 7072, Kampala, Uganda; Department of Immunology and Infection, London School of Hygiene &Tropical Medicine, London, UK; Medical Research Council/Uganda Virus Research Institute, Uganda Research Unit on AIDS, Entebbe, Uganda; School of Public Health, Faculty of Health Sciences, University of the Witwatersrand, Johannesburg, South Africa; Department of Clinical Research, London School of Hygiene &Tropical Medicine, London, UK

**Keywords:** Latent tuberculosis infection, Household contacts, Randomised design, Cytokines, Antibodies, Isoniazid preventive therapy

## Abstract

**Background:**

With the renewed emphasis to implement isoniazid preventive therapy (IPT) in Sub-Saharan Africa, we investigated the effect of IPT on immunological profiles among household contacts with latent tuberculosis.

**Methods:**

Household contacts of confirmed tuberculosis patients were tested for latent tuberculosis using the QuantiFERON®-TB Gold In-Tube (QFN) assay and tuberculin skin test (TST). HIV negative contacts aged above 5 years, positive to both QFN and TST, were randomly assigned to IPT and monthly visits or monthly visits only. QFN culture supernatants from enrolment and six months’ follow-up were analysed for *M.tb*-specific Th1, Th2, Th17, and regulatory cytokines by Luminex assay, and for *M.tb*-specific IgG antibody concentrations by ELISA. Effects of IPT were assessed as the net cytokine and antibody production at the end of six months.

**Results:**

Sixteen percent of contacts investigated (47/291) were randomised to IPT (*n* = 24) or no IPT (*n* = 23). After adjusting for baseline cytokine or antibody responses, and for presence of a BCG scar, IPT (compared to no IPT) resulted in a relative decline in *M.tb*-specific production of IFN gamma (adjusted mean difference at the end of six months (bootstrap 95 % confidence interval (CI), p-value) -1488.6 pg/ml ((−2682.5, −294.8), *p* = 0.01), and IL- 2 (−213.1 pg/ml (−419.2, −7.0), *p* = 0.04). A similar decline was found in anti-CFP-10 antibody levels (adjusted geometric mean ratio (bootstrap 95 % CI), *p*-value) 0.58 ((0.35, 0.98), *p* = 0.04). We found no effect on *M.tb-*specific Th2 or regulatory or Th17 cytokine responses, or on antibody concentrations to PPD and ESAT-6.

**Conclusions:**

IPT led to a decrease in Th1 cytokine production, and also in the anti CFP-10 antibody concentration. This could be secondary to a reduction in mycobacterial burden or as a possible direct effect of isoniazid induced T cell apoptosis, and may have implications for protective immunity following IPT in tuberculosis-endemic countries.

**Trial registration:**

ISRCTN registry, ISRCTN15705625. Registered on 30^th^ September 2015.

## Background

Adaptive immune responses to *Mycobacterium tuberculosis* (*M.tb)* infection and disease vary widely and are heterogeneous between infected individuals [1]. During the early stages of infection with tuberculosis there is a high T helper (Th) 1, and to some extent Th17, immune response to *M.tb* antigens [2, 3]. As mycobacteria multiply and *M.tb* infection progresses to disease, Th1 and Th17 responses tend to be suppressed and Th2 and regulatory responses emerge. Of note, interleukin (IL)-4, is associated with marked immunopathology and increased severity of the disease [4]. Certain *M.tb* strains, such as some subtypes of the W-Beijing strain, are associated with prominent Th2 type responses and inhibition of Th1 immune responses, and these strains are characterised by greater severity of tuberculosis infection and disease [5, 6]. The effects of IL-4 are accentuated in the presence of the Th1 cytokine tumour necrosis factor alpha (TNFα) [7, 8].

Persons with active tuberculosis also tend to have increased production of IL-10 compared to those with latent infection [9]. *M.tb* uses the lipoarabinomannan (LAM) molecule in the cell wall to induce immune regulation pathways through transforming growth factor beta (TGFβ) and regulatory T cells that suppress protective immune responses [10–12]. This same molecule, when mannosylated, interacts with mannose receptors to drive dendritic cells to release the regulatory cytokine IL-10 [13]. The IL-10 inhibits antigen processing and presentation during tuberculosis infection [14] and suppresses Th1, Th2 and Th17 function [15–18]. Thus, there is a delicate balance between pro-inflammatory immune responses that recognise *M.tb* infection, and conceal it by promoting granuloma formation, and regulatory immune responses that, while supressing Th1, Th2 and Th17 function to prevent immunopathology, may support mycobacterial replication and disease progression [18–22].

Extensive work has been carried out on treating persons with latent tuberculosis using Isoniazid Preventive Therapy (IPT). Early studies suggested a lifetime protection against active tuberculosis [23–25], but later studies have shown waning protection following short term isoniazid based regimens [26, 27]. Despite this, benefits of IPT have been demonstrated in high risk groups such as those living with HIV infection and who are latently infected with tuberculosis, where IPT reduces the risk of re-activation and progression to active tuberculosis by over 70 %, and that of mortality by 25 % [28]. Currently, the World Health Organisation (WHO) recommends the use of IPT in HIV positive individuals and children under the age of 5 years who are exposed to active tuberculosis [29].

We derived a hypothesis that household contacts of active tuberculosis patients with latent tuberculosis infection would present with mixed Th1/Th2 cytokine profiles, dominated by high concentrations of Th1 cytokines (due to recent exposure), but accompanied by Th2 and regulatory cytokine production as a result of the on-going immune modulating effects of *M.tb* infection. Regulatory profiles might be further enhanced in populations highly exposed to environmental mycobacteria, as is the case in Uganda [30].

We further hypothesised that treatment of these latently infected people with isoniazid would halt or reverse the induction of the Th2 and regulatory immune responses, causing a shift towards Th1 type immune responses. Previous studies in active tuberculosis have shown that treatment reverses the immune equilibrium from Th2 responses back to Th1 immune dominance [25, 31]. A similar effect might contribute to the long term protective effects of IPT.

We therefore initiated an exploratory trial nested in a parent cohort to investigate the effect of IPT among household contacts of active tuberculosis patients found to have latent tuberculosis infection, by comparing the immune responses among those who received six months of IPT with those who did not receive IPT.

## Methods

### Study design

This was a nested study with a randomised design within a cohort of household contacts exposed to patients with sputum positive tuberculosis in Kampala city, Uganda [32]. Participants were recruited from May 2011 to January 2012.

### Eligibility criteria for participants

Household contacts of all age groups who were exposed to sputum positive tuberculosis patients were enrolled into the larger cohort study [32]. Household contacts received HIV counselling and testing, and provided a sample of 3mls of blood for the QuantiFERON®-TB Gold In-Tube® test (Cellestis GmbH (Europe), Hannover, Germany; QFN). After blood samples were drawn from the household contacts, a tuberculin skin test (TST) was placed using the Mantoux method, using 2 Tuberculin Units (0.1 ml) of RT-23 purified protein derivative (PPD) (Statens Serum Institute, Copenhagen, Denmark), and read after 48–72 h. A positive response was a diameter of the induration area of >5 mm for HIV positive household contacts and >10 mm for those who were HIV negative [33].

The household contacts that were above the age of 5 years, were HIV negative, and tested positive on both tests, were enrolled into the nested study and followed for six months. Household contacts were excluded if they had signs and symptoms of active TB disease, liver disease or epilepsy.

### Intervention and investigations

Eligible household contacts were allocated sequential numbers at enrolment and a computer- generated list was used to randomly assign them to receive either IPT and monthly visits or monthly visits only. Household contacts in the IPT arm were offered isoniazid (5 mg/kg to a max of 300 mg) plus pyridoxine 25 mg daily for six months, which was self-administered. Neither the participants nor the care providers were blinded to which household contact received or did not receive IPT. Treatment of HIV negative, latently infected (TST/QFT positive), household contacts above the age of 5 years is neither Ugandan nor WHO policy, justifying not treating the control group. Household contacts who were HIV positive or under 5 years of age were offered routine IPT (5 mg/kg to a max of 300 mg) plus pyridoxine 25 mg daily for six months, which was self-administered, irrespective of their latent tuberculosis status, according to the World Health Organisation guidelines [34, 35].

Baseline questionnaires were administered to the household contacts and data on socio-demographics, household characteristics, TB related risk factors and exposures, medical history, and clinical findings were collected. Monthly data on clinical symptoms suggestive of active tuberculosis and possible side effects of isoniazid were collected. At the end of the six month follow up period, both the QFN and TST were repeated.

QFN supernatants from the household contacts were analysed for cytokine responses using an 11-analyte Bio-Plex Pro™ human cytokine bead array (Bio-Rad, Richmond, USA) consisting of interferon gamma (IFNγ), IL-2, TNFα, IL-4, IL-5, IL-13, IL-10, IL-17a, IL-17f, IL-21, and IL-22. The QFN assays is a 24 h whole blood stimulation assay using *M.tb* specific proteins (early secreted antigenic target 6 (ESAT-6), culture filtrate protein 10 (CFP-10), TB7.7) which have better specificity than using BCG or PPD stimulated whole blood assays. These cytokines were measured from the nil (no antigen) and TB antigen (ESAT-6, CFP-10 and TB7.7 (p4)) tubes for each household contact. All the cytokine results were within the linear range of the assay. Detection limits were as follows: IFNγ (92.6-52,719 pg/ml), IL-2 (2.1-17,772 pg/ml), TNFα(5.8-95,484 pg/ml)), Th2 cytokines (IL-4 (2.2-3,467 pg/ml), IL-5 (3.1-7,380 pg/ml), IL-13 (3.7-3,137 pg/ml)), regulatory cytokine (IL-10 (2.2-8,840 pg/ml)), and Th17 cytokines (IL-17a (4.9-12,235 pg/ml), IL-17f (3.04-18,668 pg/ml), IL-21 (8.97-147,023 pg/ml), IL-22 (3.88-11,917 pg/ml). Data were acquired using the Bio-Rad Luminex reader using Bio-Plex manager 4.1 software.

*M.tb*-specific antibody concentrations were analysed using an in-house IgG ELISA assay on baseline and end of follow up QFN culture supernatant samples from the nil (no antigen), antigen and mitogen tubes. The experiments used Immulon® 4 HBX microtitre plates (Thermo Scientific, USA) which were coated with 10 μg of PPD, CFP-10, ESAT-6 antigens per well and incubated overnight at 4 °C. The CFP-10 and ESAT-6 antigens were provided by BEI Resources which is an initiative of the National Institute of Allergy and Infectious Diseases and PPD from Statens Serum Institute, Copenhagen, Denmark. Plates were washed four times with Phosphate-Buffered Saline (PBS; pH 7.4) containing 0.05 % Tween 20 (PBS-T). Each plate was then coated with eight two-fold serial dilutions of human IgG reference standard at a top concentration of 0.625 μg/ml. Plates were blocked with 1 % skimmed milk in PBS-T for 2 h at room temperature. A dilution of 1 in 100 was made of each sample in PBS-T with 0.1 % skimmed milk (assay buffer) after which 50 μl was added to antigen-coated wells. After an incubation of 2 h at room temperature, the wells were washed as before and incubated with 50 μl/well polyclonal rabbit anti-human IgG conjugated with horseradish peroxidase (Dako, Denmark) at 0.5 μg/ml for another hour at the room temperature. Plates were then washed and enzyme activity detected by incubation with 100 μl/well *o*-phenylenediamine (Sigma-Aldrich, USA) containing hydrogen peroxide for 15 min. The reaction was stopped by addition of 25 μl/well 2 M sulphuric acid and thereafter the optical density (OD) measured at test wavelength 490 nm and reference wavelength 630 nm using an ELISA plate reader (BioTek Instruments, USA).

A pooled sample of unstimulated QFN culture supernatants from 40 active TB cases was included on each plate as a positive control for the assay. All samples and positive controls were assayed in duplicate to minimise effects of random variations in volumes due to pipetting errors. The ODs from the human IgG standard concentrations on every plate were used to generate standard reference curves for use in conversion of sample ODs to antibody concentrations in μg/ml. The concentrations of the control wells were subtracted from the test antigen wells. The net concentration was converted to ng/ml for analysis and presentation of the results.

### Outcomes

The primary outcome measures were (1) net cytokine responses and (2) *Mtb* specific antibody concentrations at the end of six-months follow up. The secondary outcomes assessed were spontaneous cytokine responses at the end of six-months follow up, possible side effects due to the IPT, and the effect of IPT on latent tuberculosis infection evidenced by changes in TST and QFN test reactions at the end of six-months follow up.

We defined spontaneous cytokine production as the cytokine concentration measured in the nil (no antigen) tube and net cytokine production as the concentration obtained after subtracting the spontaneous cytokine production from the amount produced in the TB antigen stimulated tube (antigen tube).

### Statistical methods

This was an exploratory study and the numbers enrolled were based on availability and not on a planned sample size. Data was analysed using STATA package version 12. Characteristics of participants in the two arms were compared using simple frequency tables. Cytokine responses and antibody concentrations showed skewed distributions and were described using medians and interquartile ranges. Line graphs were plotted to show the change in cytokine response and antibody concentration between baseline and end of follow up.

Each outcome measure was compared between treatment arms by fitting a multiple regression model with terms for treatment arm, baseline level of the cytokine or anti-body to control for individual variability, and presence of a BCG scar which varied between the two treatment arms and was associated with cytokine responses in this cohort [32]. The residuals from the fitted models were examined for evidence of non-constant variance, and a normal probability plot was used to determine whether the assumption of normality was reasonable. For cytokine responses, IFNγ and IL-2 met the above criteria. For other cytokines there was little evidence of non-constant variance, but several showed a departure from normality. Therefore, for each cytokine, the models were refitted with bootstrapping used to estimate the standard errors. The bootstrapping instructions were set at a random-number seed of 468 with 1000 replications.

For the antibody concentrations, there was evidence of non-constant variance, therefore a log transformation to base 10 was carried out and the results interpreted on a multiplicative scale. Because, in addition, there was still evidence of non-normality after the log transformation, bootstrapping was used to estimate the standard errors in the transformed model. Each cytokine and antibody was analysed separately.

### Ethics approval

Both the Makerere University College of Health Sciences Ethical Review Board and Uganda National Council for Science and Technology approved the study. All adult household contacts gave written informed consent to participate in the study. Parents, next of kin, caretakers or guardians gave written informed consent on behalf of the minors or children to participate in the study. In addition, children between 10 and 17 years also gave written informed assent to participate in the study.

## Results

### Study participants

**Fig. 1:**
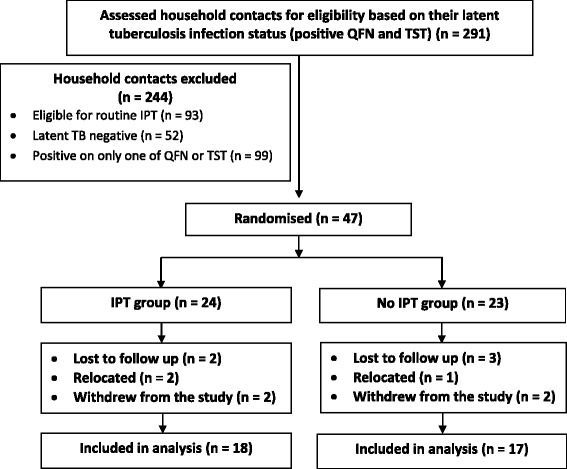
Flow diagram showing the recruitment and follow up process

Of the 291 household contacts recruited at baseline, 47 (16 %) household contacts were eligible for randomisation and had positive results for both QFN assay and TST. Two hundred and forty four household contacts were excluded as follows: 93 (38 %) were eligible for routine IPT (children under 5 years and persons with HIV infection), 52 (21 %) were negative for latent tuberculosis infection (negative QFN and TST results), and 99 (41 %) were positive on either QFN or TST but not both. Figure 1 details the rest of the recruitment, randomisation, and follow up process. The baseline demographic and exposure characteristics were similar in both arms except for differences in the frequency of presence of a BCG scar, and in the baseline cytokine concentrations. The Th1 type cytokines had the highest concentrations at baseline compared to Th2, regulatory, and Th17 cytokines (Table 1).

**Table 1:** Baseline characteristics of the household contacts with latent tuberculosis infection that were randomised to either the no IPT or IPT arms

Variable	Level	No IPT (*n* = 23) n (%)	IPT (*n* = 24) n (%)
Household contact relating factors			
Sex	Female	16 (70)	15 (63)
	Male	7 (30)	9 (37)
Age (median)		25	24
Age group	6–12	3 (13)	4 (17)
	13–18	3 (13)	3 (13)
	>18	17 (74)	17 (70)
Socio-economic status	Lower	11 (48)	10 (42)
	higher	12 (52)	14 (58)
Relationship to index case	First degree relative	18 (78)	20 (83)
	Not first degree relative	5 (22)	4 (17)
Proximity to index case	Shared meals with index case	0	2 (8)
	Cared for index case	7 (30)	12 (50)
	Shared room with index case	12 (52)	6 (25)
	Shared bed with index case	4 (18)	4 (17)
Duration of contact with index case	>6 h/day	22 (96)	20 (83)
	<6 h/day	1 (4)	4 (17)
Smoking	No	18 (78)	20 (83)
	Yes	5 (22)	4 (17)
Alcohol	No	15 (65)	17 (71)
	Yes	8 (35)	7 (29)
BCG scar	No	12 (52)	7 (29)
	Yes	11 (48)	17 (71)
^a^Baseline cytokine responses (medians (IQR)) pg/ml	IFNγ	1201.1 (336.8–2246.6)	648 (259.6–2605.4)
	TNFα	1 (1–11.4)	6.8 (1–74.6)
	IL-2	339.8 (138.4–415.2)	291.2 (80.6–444.4)
	IL-5	2.1 (1–9.9)	1 (1–1)
	IL-13	21.9 (2.3-35.3)	2.1 (1–10.6)
	IL-10	1 (1–1)	1 (1–1)
	IL-17a	1 (1–1)	1 (1–1.8)
	IL-17f	3.8 (1–6.7)	1 (1–1.9)
	IL-21	1 (1–1)	1 (1–1)
	IL-22	1.1 (1–12.4)	1 (1–4.9)
*M.tb* specific antibody responses	PPD	5200 (2950–8800)	5550 (3250–8200)
(medians (IQR)) ng/ml	CFP-10	3100 (1500–4800)	2700 (2050–4900)
	ESAT-6	5000 (2400–7900)	3700 (2550–5600)
Index case relating factors			
HIV status of index case	Negative	20 (87)	18 (82)
	Positive	3 (13)	4 (18)
Sputum positivity	1+	4 (17)	7 (29)
	2+	10 (43)	4 (17)
	3+	9 (40)	13 (54)
Duration of Index patient cough (weeks)	0–4	6 (26)	7 (29)
	5–14	9 (39)	7 (29)
	15–24	4 (17)	8 (33)
	>25	4 (17)	2 (8)

^a^Baseline cytokine responses were obtained from QFN culture supernatants and are represented as net (antigen stimulated minus unstimulated) values

Over 70 % of the household contacts in each arm completed the study. The main reason for loss to follow up was a change in the address of the household contacts. The household contacts that withdrew from the study were unable to continue with the follow up because of their busy schedules. All received six months of IPT. There were no documented side effects of isoniazid treatment and there was no incidence of active tuberculosis.

### Effect of IPT on cytokine responses

**Fig. 2:**
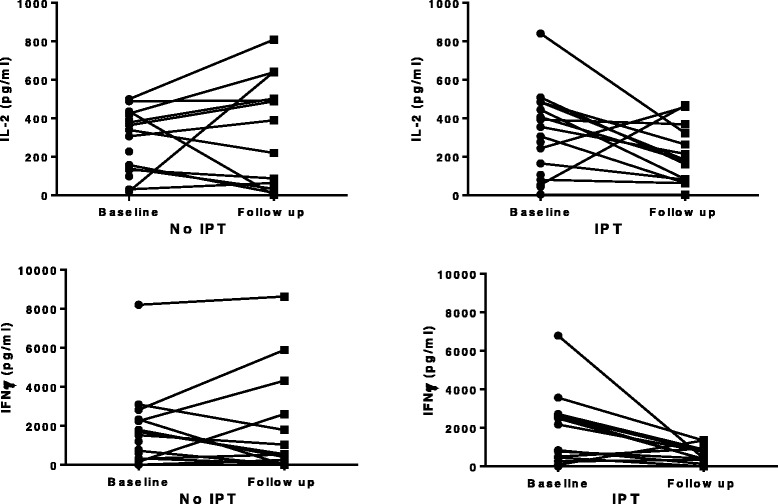
Comparison of the change in *M.tb*-specific net IFNγ and IL-2 cytokine production between enrolment and after 6 months of follow up in the no IPT or IPT treatment arms. Net cytokine production (after subtraction of spontaneous cytokine production) was determined in response to TB antigens (ESAT-6, CFP-10 and TB7.7 (peptide 4)) in QFN supernatants tested for cytokine content using multiplex bead array between baseline and end of six months among the household contacts with latent tuberculosis infection

This study was exploratory, comparing the effect of IPT on net cytokine responses and antibody concentrations at the end of follow up as the primary outcomes of interest, among latently infected contacts who were above 5 years of age and were HIV negative. We observed a drop in the *M.tb*-specific IFNγ and IL-2 cytokine production in the IPT arm (median (IQR)) from 648.3 pg/ml (259.5–2605.4) to 412.3 pg/ml (129.0–878.1), and from 291.1 pg/ml (80.6–444.3) to 179.8 pg/ml (62.5–322.4) respectively, but no change or a slight rise in the no IPT arm (Fig. 2).

The multiple linear regression models confirmed that IPT caused a relative decline in the same Th1 cytokine responses (Table 2). After adjusting for baseline cytokine responses and presence of a BCG scar, there was a lower net production of IFNγ in the IPT arm (adjusted mean difference at the end of six months (bootstrap 95 % confidence interval, p-value) -1488.6 pg/ml ((−2682.5, −294.8), *p* = 0.01), and of IL- 2 (−213.1 pg/ml (−419.2, −7.0); *p* = 0.04). However, IPT had no effect on the Th2, regulatory and Th17 cytokine profiles (Table 2). IPT had no effect on spontaneous cytokine production at the end of six months.

**Table 2:** The impact of isoniazid preventive therapy on the net cytokine responses at the end of six-months follow up among household contacts with latent tuberculosis infection

Cytokine	Mean at end of six months	Mean at end of six months	Adjusted mean difference at the end of six months^a^	*P*-value
	(pg/ml; 95 % CI)	(pg/ml; 95 % CI)	(pg/ml; 95 % CI)	
	No IPT (*n* = 17)	IPT (*n* = 18)		
IFN-γ	2003.6(372.7, 3634.4)	541.1 (290.8, 791.3)	−1488.6(−2682.5, −294.8)	0.01
IL-2	337.0(167.3, 506.8)	193.9 108.5, 279.3)	−213.1(−419.2, −7.0)	0.04
TNF-α	34.4(−10.6, 79.4)	55.6(−6.3, 117.6)	34.1(−40.0, 108.2)	0.36
IL-5	15.5(−3.8, 34.9)	1.1(0.8, 1.4)	−21.2 (−53.9, 11.3)	0.20
IL-13	35.4(−3.2, 74.2)	5.1(0.9, 9.4)	−45.4(−104.4, 13.4)	0.13
IL-10	1.0(0.9, 1.1)	3.3(−0.8, 7.6)	2.0 (−1.43, 5.4)	0.25
IL-17a	2.4(−0.4, 5.2)	1.5(0.7, 2.3)	0.4 (−0.4, 1.2)	0.33
IL-17f	12.3(3.3, 21.3)	10.2(−6.1, 26.6)	−5.8(−17.5, 5.7)	0.32
IL-21	34.0(−22.7, 90.7)	123.8(−133.9, 381.6)	−11.6(−40.9, 17.5)	0.43
IL-22	27.0(1.3, 52.7)	6.1(−0.2, 12.6)	−6.8(−88.8, 75.0)	0.86

^a^This is the expected value of the mean difference in cytokine level at six months, comparing participants who received IPT to those did not receive IPT, adjusting for the baseline level of the cytokine and the BCG scar status. Each cytokine was analysed in a separate model, with bootstrapping to estimate the confidence intervals

### Effect of IPT on M.tb specific antibody concentrations

**Fig. 3:**
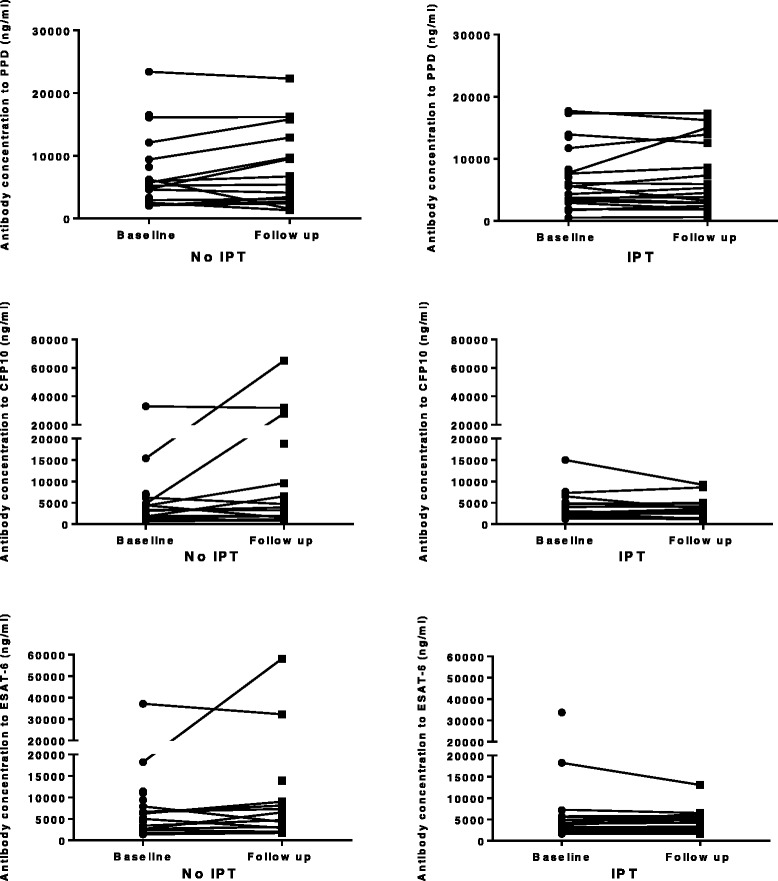
Comparison of the change in *M.tb-*specific antibody production between enrolment and after 6 months of follow up in the no IPT or IPT treatment arms. Antibody concentrations were determined in response to TB antigens (PPD, CFP-10 and ESAT-6) in QFN supernatants using an IgG ELISA assay among the household contacts with latent tuberculosis infection

We assessed the effect of IPT on IgG antibody responses to PPD, CFP-10, and ESAT-6 and found that there was no major change in the absolute antibody concentrations in either arm (Fig. 3). However, after adjusting for baseline antibody concentration and presence of a BCG scar, we observed that IPT caused a relative decline in the antibody concentrations to CFP-10 (adjusted geometric mean ratio (bootstrap 95 % CI), p-value) 0.58 (0.35, 0.98), *p* = 0.04. There was no effect on antibodies to ESAT-6 or PPD (Table 3).

**Table 3:** The impact of isoniazid preventive therapy on the net log10 transformed *M.tb* specific-antibody concentration at the end of six months follow up among household contacts with latent tuberculosis infection

Antibody	Geometric Mean at the end of six months	Geometric Mean at the end of six months	Adjusted geometric mean ratio (bootstrap 95 % CI)^a^	*P*-value
	No IPT (*n* = 17)	IPT (*n* = 18)		
PPD	3.7(3.5, 4.0)	3.6(3.4, 3.8)	1.03(0.76, 1.40)	0.82
CFP-10	3.6(3.3, 3.9)	3.4(3.3, 3.5)	0.58(0.35, 0.98)	0.04
ESAT-6	3.6(3.4, 3.9)	3.5(3.4, 3.7)	0.87(0.65, 1.18)	0.60

^a^This is the ratio of the expected value of the geometric mean antibody level at six months comparing participants who received IPT to those who did not receive IPT, adjusting for the baseline level of the antibody and the BCG scar status. Each antibody was analysed in a separate model

### Effect of IPT on tuberculosis infection

We assessed the effect of IPT on *M.tb* infection by investigating the number of TST and QFN reversions found at the end of six months of follow up. There was only one QFN test reversion and it occurred in the IPT arm. There were 26 TST reversions: 15/18 (83 %) of participants assessed in the IPT arm showed TST reversions and 11/17 (65 %) in the no-IPT arm (*p* = 0.26, Fisher’s exact test). Among reverters, the average TST diameter fell from 14 mm to 2 mm in the IPT arms and from 18 mm to 4 mm in the no IPT arm.

## Discussion

In this study, we found that the household contacts of active tuberculosis patients who had evidence of latent *M.tb* infection had strong *M.tb*-specific Th1 cytokine responses at baseline. Contrary to our hypothesis, we found low production of Th2 cytokines which supports evidence that marked Th2 cytokine responses are markers of TB disease severity rather than of latent infection [4, 31]. In addition, we found very low production of the regulatory cytokine IL-10 and therefore a remote possibility of on-going immune modulation by *M.tb* in these contacts. In this study we used supernatants from the QFN assay to measure the different cytokines. This assay is designed to measure IFNγ release and may therefore not be optimal for measuring other cytokines including IL-10.

Some studies have also demonstrated that healthy contacts with stable latent tuberculosis infection present with high Th1 and low Th2 cytokine profiles caused by up-regulation of the IL-4 antagonist IL-4δ2 [2, 36]. Immune responses in people with latent tuberculosis vary with the different stages of infection [1, 37] and may also be influenced by prior exposure to environmental bacteria [30], the time since infection with *M.tb* [38], the infective dose of *M.tb* [19], and the dynamic status between controlling infection and progressing to disease [39]. Prior exposure with BCG immunisation at birth can also be an attributable factor to the sustained high Th1 responses in this population [40]. Our baseline findings are consistent with recent low-dose exposure to *M.tb* infection among contacts with an effective, immune controlling response to the *M.tb* infection [41]. This is supported by the high rate of reversions of the TST even in the no IPT arm, and lack of progression to active TB.

At the end of six months, we found that IPT led to a decrease in the levels of Th1 cytokines and not of the Th2 or regulatory cytokines as anticipated. Reduction in IFNγ cytokine responses following IPT has been demonstrated before [38, 42–45]. One possible explanation is effective clearance of dormant *M.tb* by isoniazid, leading to a decline in circulating effector cells and leaving only long-lived memory T cells which secrete low Th1 cytokine concentrations on re-exposure to *M.tb* antigens [46]. Added to this, it is possible that the presence of BCG bacilli from previous immunisation at birth sustains expression of CD4 effector memory cells, and that clearance of BCG bacilli by isoniazid therapy reduces the number of these cells [40]. A third possibility is a reduction in *M.tb* specific IFNγ and IL-2 producing T-cells [42, 47] due to isoniazid induced apoptosis [48] however, this data has only been generated in one animal study. There have been some concerns about the reduction of *M.tb* related cytokine responses that may be protective after treatment with isoniazid: this might increase the risk of *M.tb* re-infection or re-activation, in settings with high exposure to *M.tb* [26, 27, 49]. Some studies have addressed this by prolonging the duration of IPT [50, 51]. The incidence of acquiring active TB following IPT or no IPT in these trials was similar in the post IPT period, however these trials were in HIV patients who are highly susceptible to acquiring active TB disease. Effects might be different in HIV negative individuals.

We found that IPT caused a relative decline in the antibody concentration to CFP-10 between baseline and follow up, which was similar with the anti-ESAT concentrations though the effect on the latter was statistically weak. There was no effect of IPT on antibody concentrations to PPD. The effect of treatment of active tuberculosis on *M.tb*-specific antibody responses is variable with some studies showing a decrease [52, 53], while others a sustained increase up to 2 years following chemotherapy [54, 55]. In *M.tb* infection, CFP-10 and ESAT-6 secretory antigens form a complex which is involved in mycobacterial virulence [56]. The fact that the decline in *M.*tb-specific antibody responses was only seen with CFP-10 reflects an effect of IPT on *M.tb* specific responses. This could be explained by antibody involvement in clearing mycobacterial antigens by formation of antigen-antibody complexes [57], though this may be more pronounced for antigens such as LAM [58].

The PPD responses on the other hand may not only be *M.tb* specific but could be due to exposure to environmental mycobacteria or non-tuberculosis mycobacteria (NTM). Also, even if the IPT led to a reduction in PPD responses that are “*M.tb* specific”, this effect could be “drowned” by the PPD responses induced as a result of continuous exposure by NTM encountered in the environment. We have previously shown high antibody titres to PPD that were maintained for up to 40 years after BCG immunisation [59]. The majority of our household contacts were immunised with BCG and this previous exposure (as well as prior exposure to environmental mycobacteria) may have contributed to the sustained antibody concentrations to PPD. It is also possible that IPT led to mycobacterial killing and release of antigens resulting in sustained antibody production to PPD while, on the contrary, reducing exposure to the antigens secreted by living organisms. Similar to T cell based responses, antibody responses vary with the type of antigen, stage and bacterial load of *M.tb* infection [60]. A study with a longer follow up duration would be beneficial to determine any later change in the PPD antibody concentrations.

To assess the effect of IPT on reduction of tuberculosis infection, we analysed the change in QFN and TST tests at the end of follow up. There was only one QFN reversion, and this occurred in the IPT arm, similar to another study conducted by Johnson, et al., in South Africa [43]. In our study, TST reversion was more common in the IPT arm than in the no IPT arm, but this was a weak difference. Johnson, et al.*,* found that it was common to obtain TST reversions following IPT [41], and the average TST diameter for those that reverted changed from 16.3 mm to 1.4 mm after IPT, again similar to our study. There is still contradictory evidence regarding the effect of IPT on TST reactions. The earlier Public Health Service trials reported in North America showed that reversions of the TST reactions were as frequent in the placebo groups as in the isoniazid groups, in keeping with our results [25].

This exploratory study included small numbers of subjects in each IPT treatment arm giving us limited power to draw solid conclusions. Apparent effects may have occurred by chance, and real effects may have been missed. For example, the TST reversion rate appears to be higher in the IPT arm, but the p-valve was not statistically significant. The small sample size resulted mainly from our strict eligibility criteria which required a household contact to be positive on both the QFN and TST tests, yet there were a large number of household contacts that had discordant QFN and TST test results. The impact of IPT in the subgroups positive only on QFN would be of interest. A larger study would be warranted to investigate effects of IPT further. Due to the nature of the study design, we were unable to know the duration of latent tuberculosis infection for each household contact. As discussed above, this is important for the interpretation of our findings [1]. Placing a TST can influence subsequent immune responses to mycobacterial antigen [61] but this is unlikely to have biased our results since samples for QFN were taken before the TST was placed at both time points, and all participants had the same TST exposure. We were unable to satisfactorily monitor for compliance to the IPT. However, the observed differences between the two arms are consistent with an effect of treatment among the participants who were provided with the drug.

## Conclusions

These findings provide further evidence that there is an effect of IPT on the Th1 cytokine responses to *M.tb-*specific antigens, and the *M.tb-*specific antibody concentration, particularly to CFP-10, but gives no evidence of an effect on the Th2, regulatory and Th17 cytokine profiles, or on *M.tb-*specific antibody concentrations to PPD or ESAT-6. This could reflect a reduction in the mycobacterial burden, and hence be taken as a positive response to treatment, promoting the desired outcome of disease prevention. Alternatively, these results might perhaps be a direct effect of the drug. The implications of the decline in T cell and antibody responses are not clear, given that correlates of protection against tuberculosis are not well defined [62, 63]. Longer follow up studies have been carried out, mainly in the USA, which showed that the risk of subsequent progress to active TB disease reduced with IPT [24, 25]. We lack information from similar studies carried out in highly TB-endemic settings, where re-exposure is very common. Could a reduction in potentially protective immune responses increase vulnerability upon re-exposure, possibly out-weighing the benefit of preventing reactivation of latent infection? In such settings, there might be value in a larger study investigating the risks and benefits of short term IPT for low risk household contacts of patients with active TB.

## Abbreviations

CFP-10: Culture filtrate protein 10
ESAT 6: Early secreted antigenic target-6
IFNγ: Interferon gamma
IL: Interleukin
LAM: Lipoarabinomannan
M.tb: Mycobacterium tuberculosis
OD: Optical density
PBS-T: Phosphate-buffered saline with Tween 20
PPD: Purified protein derivative
QFN: QuantiFERON®-TB Gold In-Tube
TGFβ: Transforming growth factor beta
Th: T helper
IPT: Isoniazid preventive therapy
TNFα: Tumour necrosis factor alpha
TST: Tuberculin skin test
